# An Innovative Approach to Control *H. pylori*-Induced Persistent Inflammation and Colonization

**DOI:** 10.3390/microorganisms8081214

**Published:** 2020-08-10

**Authors:** Paola Cuomo, Marina Papaianni, Andrea Fulgione, Fabrizia Guerra, Rosanna Capparelli, Chiara Medaglia

**Affiliations:** 1 Department of Agricultural Sciences, University of Naples Federico II, 80055 Portici, Naples, Italy; paola.cuomo@unina.it (P.C.); marina.papaianni@unina.it (M.P.); 2 Istituto Zooprofilattico Sperimentale del Mezzogiorno (IZSM), 80055 Portici, Naples, Italy; andrea.fulgione@unina.it; 3 Department of Pharmacy, University of Naples Federico II, 80131 Naples, Italy; fabrizia.guerra@unina.it; 4 Department of Microbiology and Molecular Medicine, University of Geneva Medical School, 1211 Geneva, Switzerland; chiara.medaglia@unige.ch

**Keywords:** *Helicobacter pylori*, bacteriophages, inflammation

## Abstract

*Helicobacter pylori* (*H. pylori*) is a Gram-negative bacterium which colonizes the human stomach. The ability of *H. pylori* to evade the host defense system and the emergence of antibiotic resistant strains result in bacteria persistence and chronic inflammation, which leads to both severe gastric and extra-gastric diseases. Consequently, innovative approaches able to overcome *H. pylori* clinical outcomes are needed. In this work, we develop a novel non-toxic therapy based on the synergistic action of *H. pylori* phage and lactoferrin adsorbed on hydroxyapatite nanoparticles, which effectively impairs bacteria colonization and minimizes the damage of the host pro-inflammatory response.

## 1. Introduction

*Helicobacter pylori* (*Hp*) is a Gram-negative bacterium able to induce chronic infections in humans. It colonizes the gastric mucosa of over 50% of the population worldwide. *Hp* infects gastric epithelial cells and promotes chronic inflammation, leading to chronic gastritis, which can eventually degenerate in peptic ulcer and gastric carcinoma [1,2,3]. *Hp* also interferes with biological processes outside the stomach, causing extra-gastric diseases such as cardiovascular, neurological and metabolic diseases [4,5]. Our recent works suggest *Hp*-induced persistent inflammation as the key factor responsible for the pathogenicity of extra-gastric diseases (Papaianni et al., manuscript in preparation; Fulgione et al., under submission). Therefore, the clinical outcome of *Hp* infection is determined by the severity of local and systemic inflammatory response, which is regulated by both host and pathogen factors, rather than by the bacterial infection itself. More specifically, *Hp* virulence factors and their ability to evade the host immune system, especially in immunocompromised individuals, trigger an uncontrolled inflammatory response. Furthermore, the *Hp* genetic plasticity and the inappropriate use of antibiotics favor the spread of antibiotic resistant strains, which are difficult and sometimes impossible to treat [6].

Given this scenario, therapeutic treatments for *Hp* infection able to overcome the antimicrobial resistance and mitigate the inflammatory response represent a valid alternative to common antibiotic therapies. Phages are considered a promising substitute for traditional antibiotics, thanks to their unique characteristics such as host specificity and narrow spectrum of activity resulting in no alteration of gut microbiota, exponential reproduction, and safety [7,8]. Indeed, phages are significantly better tolerated than antibiotics, as they replicate only in the target bacterium but cannot infect mammalian cells or interfere with their molecular mechanisms [9]. Moreover, the ability of phages to inhibit the inflammatory response favors the success of phage therapy [10,11], whose applicability to *Hp* infection is still poorly studied. Of note, bacteria also develop resistance to phages, but it is incomparably easier to develop new phages than new antibiotics [12]. The application of phages to treat bacterial infections involving different body sites is widely documented. The majority of these studies report the beneficial effects of phage administration for topical treatment of localized skin bacterial infections in wounds, burns, and trophic ulcers, including diabetic foot ulcers [13].

Despite its numerous advantages, the applicability of phage therapy to *Hp* infection is hindered by the harsh physiological conditions of the gastric environment. Specifically, the acidity of the gastric juice together with the digestive enzymes dramatically alter both the biological and structural components of phages, thus reducing their proliferation and concentration at the site of the infection [14,15]. The high sensitivity of phages to an acidic environment [16] can be overcome by natural coating, which protects phage particles from the gastric acidic environment, thus enhancing their stability without affecting the phage’s infection ability. Natural coating thus represents an effective strategy to extend the applicability of phage therapy to gastric infections.

For many years, due to the development of medical nanotechnologies, nanoparticles have been studied and applied in the pharmaceutical field as drug delivery, in order to improve drug pharmacokinetic and pharmacodynamic [17,18]. Hydroxyapatite (HA) Ca_10_(PO_4_)_6_OH_2_ is an inorganic material that is present as a natural component of human teeth and bones. Biocompatibility and biological inertia, as well as nanotechnology development [19], make HA the scaffold of choice for drug delivery [20].

Recent studies have shown the significant contribution of HA when complexed with bacteriophages. In particular, the complex phage-HA reduces the high susceptibility of phages to the acid gastric environment and increases its stability, resistance, and lytic activity [21,22].

In addition to phage therapy, another valid approach could be represented by the use of antimicrobial molecules such as the lactoferrin, whose activity against *Hp* infection has been widely investigated [23]. Lactoferrin (LF) is a mammalian glycoprotein present in milk and other mucosal secretions [24,25]. Due to its location, LF can be considered as a first-line mucosal defense peptide, which contributes to host protection against invading pathogens. Similar to phages, LF can act both on the bacteria (direct microbicidal and microbiostatic action) or can modulate the inflammatory response (indirect action). Moreover, it has been shown that, when complexed with HA, LF biological properties are increased [26].

In the present study, we evaluated the antimicrobial activity of a lytic bacteriophage specific to (Hp φ) alone or combined with LF adsorbed on HA nanoparticles (Hp φ +LF-HA) against *Hp* infection. Interestingly, we demonstrated the synergistic action of phage and lactoferrin in reducing *Hp*-induced inflammation, in addition to *Hp* colonization.

## 2. Materials and Methods

### 2.1. Bacteria

Bacteria used in this study were isolated from human gastric biopsies, kindly provided by the gastroenterology department of “Ospedale Evangelico Villa Betania”, Naples. The *Hp* species was confirmed by PCR assay of the specific pathogen gene glmM [27]. Bacteria were grown in Brain Heart Infusion broth (BHI; Oxoid, Thermo Fisher Scientific, Waltham, MA, USA), supplemented with horse serum 10% at 37 °C and 5% CO_2_ until they reached the exponential growth phase (OD_600_: 0.6 to 0.8). Then, they were harvested by centrifugation (8 × 10^3^ g for 10 min), washed with saline solution (0.15 M NaCl) and resuspended in BHI broth or saline solution (10^6^–10^8^ CFU/mL).

### 2.2. Phage Isolation

Samples from different patients were grown in BHI broth supplemented with horse serum 10% at 37 °C and 5% CO_2_. When cultures reached the exponential growth phase (OD_600_: 1.5 to 1.8), bacteria were washed with BHI broth supplemented with horse serum 10% and incubated again for 4 h at 37 °C. The supernatants were filtered through a 0.45 µm membrane and screened for the presence of phages by the spot test [28]. Supernatants, which resulted positive in the spot test, were tested five times again by performing the plaque-forming assay [21]. Individual plaques were expanded in BHI broth supplemented with horse serum 10% (2 mL) containing the sensitive bacterial host (10^6^ CFU). Phage purification was carried out as described previously [29].

### 2.3. Adsorption Rate, Latent Period, and Phage Burst Size

The adopted procedures were those described previously [30]. Briefly, to measure the adsorption rate, 1 mL phage Hp φ (1.5 × 10^3^ PFU/mL) and 1 mL *Hp* (5 × 10^8^ CFU/mL) were mixed and the number of free phage particles was determined after treatment with chloroform (200 µL). To determine the latent period and burst size, *Hp* bacteria (5 × 10^8^ CFU/mL) were incubated with phage Hp φ (3 × 10^3^ PFU/mL) for 5 min, washed with cold BHI broth to remove free phage particles, and then resuspended in fresh medium. The cell suspension was periodically titrated for newly produced phage on *Hp* lawn [31].

### 2.4. Complex Hp Phage, Lactoferrin, and Hydroxyapatite Nanoparticles (Hp φ +LF-HA)

The Hp φ +LF-HA complex was prepared by mixing the LF-HA previously described (Fulgione et al., 2016) with 1 mL of Hp φ (10^8^ PFU/mL) and incubated—under shaking condition—at room temperature for different times (0, 30′, 90′, 180′, 300′ and 24 h). After the different incubation all the samples were centrifuged, the pellet was suspended in distillate water, and the supernatant stored. The concentration of the active phage particles in the pellet was evaluated by the double layer assay (DLA) method [28]. Specifically, after an overnight incubation the phage particles were calculated. The samples that displayed the highest activity with the lower concentration were selected as optimal incubation time. Concurrently, the supernatant was tested for detecting the amount of phage tied to the LF-HA. Briefly, the supernatant was spotted (three spots of 10 µL) on the soft agar overlay. After overnight incubation, the titer was determined as reported by Papaianni et al. [28].

### 2.5. Measurement of Cell Viability

#### 2.5.1. MTT Assay

Analysis of cell viability was performed using the CellTiter 96^®^ AQueous One Solution Cell Proliferation Assay system (MTS assay) (Promega, Madison, WI, USA) (Carrieri et al., 2017). Human Caucasian gastric adenocarcinoma (AGS) cells, grown in DMEM medium (Gibco, Scotland) supplemented with 10% fetal bovine serum (FBS), penicillin (100 IU/mL), and streptomycin (100 μg/mL) (all from Gibco, Scotland), were seeded in a 96-well plate (2500 cells/well) and incubated at 37 °C, in a humidified atmosphere with 5% CO_2_. After cells adhesion, they were treated with (1) *Hp* phage (Hp φ; 10^6^ PFU/mL); (2) lactoferrin adsorbed on hydroxyapatite nanoparticles (LF-HA; 200–600 µg/mL); (3) the complex Hp φ +LF-HA described above. Twenty μL of CellTiter 96^®^ AQueous One Solution reagent was added to each well, according to the manufacturer’s instructions. Absorbance was recorded at 490 nm after 2 h using an EnVision 2102 multilabel reader (PerkinElmer, Waltham, MA, USA).

#### 2.5.2. Trypan Blue Test

Analysis of cell viability was performed by plating AGS cells (10^6^ cells/well) in 6-well plates and letting them adhere in DMEM supplemented with 10% FBS, penicillin (100 IU/mL), and streptomycin (100 μg/mL). After adhesion cells were washed three times with phosphate buffer saline (PBS; Gibco, Thermo Fisher Scientific, Waltham, MA, USA) and then treated with (1) *Hp* phage (Hp φ; 10^5^ PFU/mL or 10^6^ PFU/mL); (2) lactoferrin adsorbed on hydroxyapatite nanoparticles (LF-HA; 200–600 µg/mL); (3) the complex Hp φ +LF-HA described above, for 24–48–72 h. At each time point, cells were washed three times with PBS and then 1.5 mL of 1% trypsin was added in each well. After incubation at 37 °C for 3 min, 3 mL of culture medium were added to each well and the whole mixture was transferred into a test tube for centrifugation and centrifuged for 3 min at 1000 g. The supernatant being aspirated and discarded, the cell pellet was resuspend with 1 mL of culture medium and 10 µL of resuspended cells were mixed with 10 µl of Trypan blue, which is able to color the necrotic cells. The percentage of viability was calculated as reported: N° viable cells/N° nonviable cells + viable cells × 100.

#### 2.5.3. NO_2_ Measurements

The accumulation of NO_2_ in culture media was determined using Griess Reagent Kit for Nitrite Determination (Molecular Probes, Boyds, MA, USA), following the manufacturer’s instructions [32].

### 2.6. SEM Image

Water suspensions of the samples Hp φ +LF-HA complex—previously centrifuged at 13.000 rpm for 15 min—were deposited on 5 × 5 mm silicon chips and the solvent was evaporated under vacuum at 30 °C. The silicon supports were mounted on 13 mm SEM aluminum stubs and sputtered with a nanometric conductive layer of Au/Pd alloy using a Desk V TSC coating system (Denton Vacuum, Moorestown, NJ, USA). SEM micrographs were recorded with a Field Emission Gun Scanning Electron Microscope (FEGSEM) Nova NanoSem 450 (FEI/ Thermo Fisher Scientific, Waltham, MA, USA) under high vacuum conditions.

### 2.7. Helicobacter Pylori Culture, Gastric Cell Infection

AGS cells were distributed in a 24-well plate (10^5^/well) and allowed to adhere (37 °C, 5% CO_2_) in DMEM supplemented with 10% FBS, penicillin (100 IU/mL), and streptomycin (100 μg/mL). Wells were washed with DMEM to remove non-adherent cells. Cells were fed with 1 mL DMEM supplemented with 10% FBS and infected or not with *Helicobacter pylori* (10^4^ CFU/well). The plates were centrifuged (1000 rpm, 10 min) to facilitate cell contact, and then incubated for 24 h at 37 °C in 5% CO_2_. Cells infected with *Hp* were washed and treated with Hp φ (10^6^ PFU/well) for 3 or 24 h at 37 °C in 5% CO_2_.

### 2.8. Quantitative Gene Expression Analysis

Total RNA was extracted following the Tryzol reagent protocol (Invitrogen, Thermo Fisher Scientific, Waltham, MA, USA). Gene transcript levels were measured using Power SYBR^®^ Green PCR Master Mix (Applied Biosystems^®^, Thermo Fisher Scientific, Waltham, MA, USA) on a QuantStudio™ 3 Real-Time PCR System (Applied Biosystems^®^, Thermo Fisher Scientific, Waltham, MA, USA) [33]. QuantStudio Design & Analysis Sofware v1.1 (Applied Biosystems Thermo Fisher Scientific, Waltham, MA, USA) was used for the analysis of gene expression. All samples were normalized to GAPDH as reference housekeeping gene. The relative quantitative expression was calculated using the 2−ΔΔ*C*T method [34].

### 2.9. ROS Detection Assay

ROS formation was assayed using dihydrorhodamine 123 (DHR) as described by Palomba et al. [35]. Briefly, AGS cells infected with *H. pylori*, loaded with DHR (10 µM for 20 min), were treated for 1 h with (1) *Hp* phage (Hp φ; 10^6^ PFU/mL); (2) lactoferrin adsorbed on hydroxyapatite nanoparticles (LF-HA; 200–600 µg/mL); (3) the complex Hp φ +LF-HA described above, and analysed with a Leica DMI6000 fluorescence microscope equipped with a Leica DFC320 cooled digital CCD camera (Leica Microsystems). The excitation and emission wavelengths were 488 and 515 nm, respectively. Images were collected with exposure times of 100–400 ms, digitally acquired, and processed for fluorescence determination at the single cell level with Metamorph Imaging Software (Leica MetaMorph © AF, Wetzlar, Germany). Mean fluorescence values were determined by averaging the fluorescence values of at least 50 cells/treatment.

## 3. Results

### 3.1. Phage Isolation

**Figure 1:**
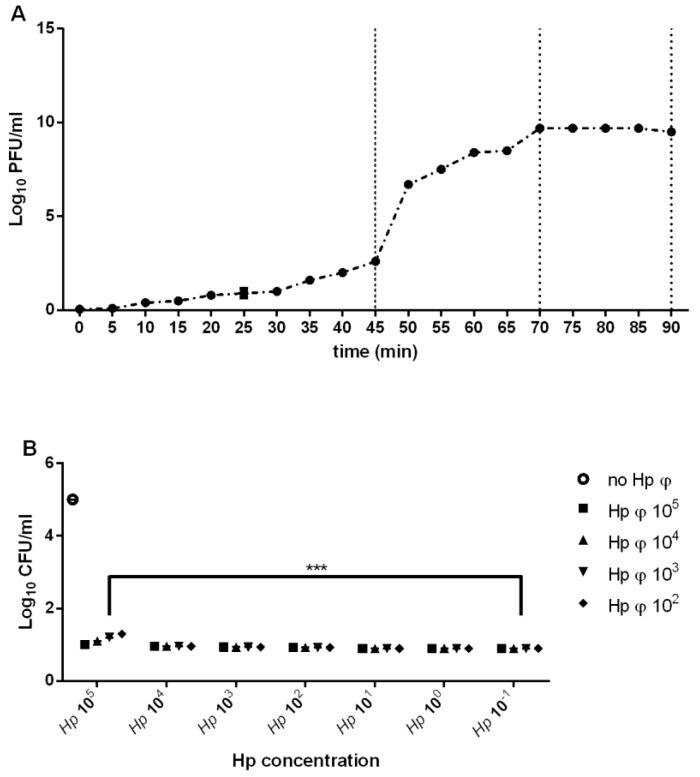
Phage characterization. (**A**) One step growth curve of phage. (**B**) Representation of phage activity on *Hp* growth. The figure illustrates the bacterial plate counts (CFU/mL) after the activity of the phage. Each value is the mean ± SD of three independent experiments. *** *p* < 0.001. Statistical analysis was performed with Student’s *t*-test. Values are expressed as the mean ± SD from three independent experiments with three replicates for each data point.

Following the treatment of the positive *Hp* isolated from different patients, several phage particles were yielded. The phage displaying the largest host range was designated Hp φ (Table 1). After the purification and the starvation Hp φ was further characterized based on the adsorption rate (1.89 × 10^9^ mL/min), latent period (45 min), and burst size (80 PFU) (Figure 1A). The phage lytic activity, analyzed by MOI test, was independent of *Hp* concentration (Figure 1B).

### 3.2. Hp φ +LF-HA Complex

**Figure 2:**
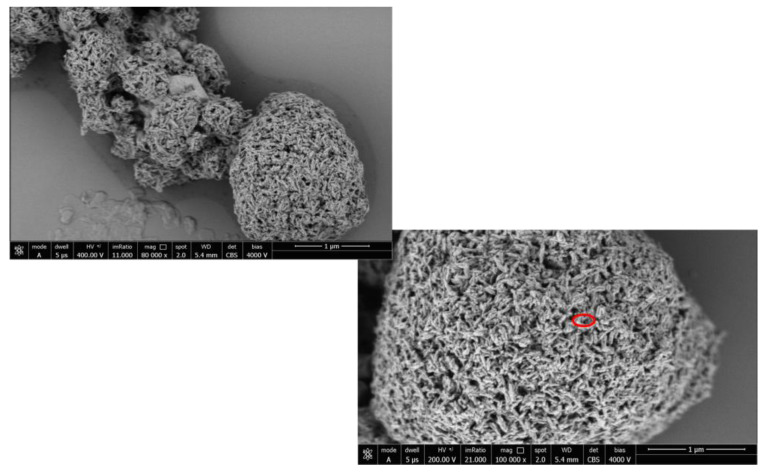
SEM image of the complex Hp φ + LF-HA. The phage’s capsid is represented as the light point (red square) on the HA nanocrystal.

The combination ratio of Hp φ and HA-LF (Hp φ +LF-HA) was determined for the following experiments. In particular, the optimization of the treatment with Hp φ and HA-LF complex was carried out by testing different concentrations of Hp φ incubated with HA-LF (data not shown). The optimal combination ratio was further characterized using the SEM microscopy analysis in order to validate the bounds of the phage with LF-HA. Previous studies have characterized the lactoferrin adsorbed onto the HA nanocrystal [23]; Figure 2 shows the phage bounds into the pores of the nanocrystal HA particles and lactoferrin.

### 3.3. Cytotoxic Activities of the Phage Hp φ

**Figure 3:**
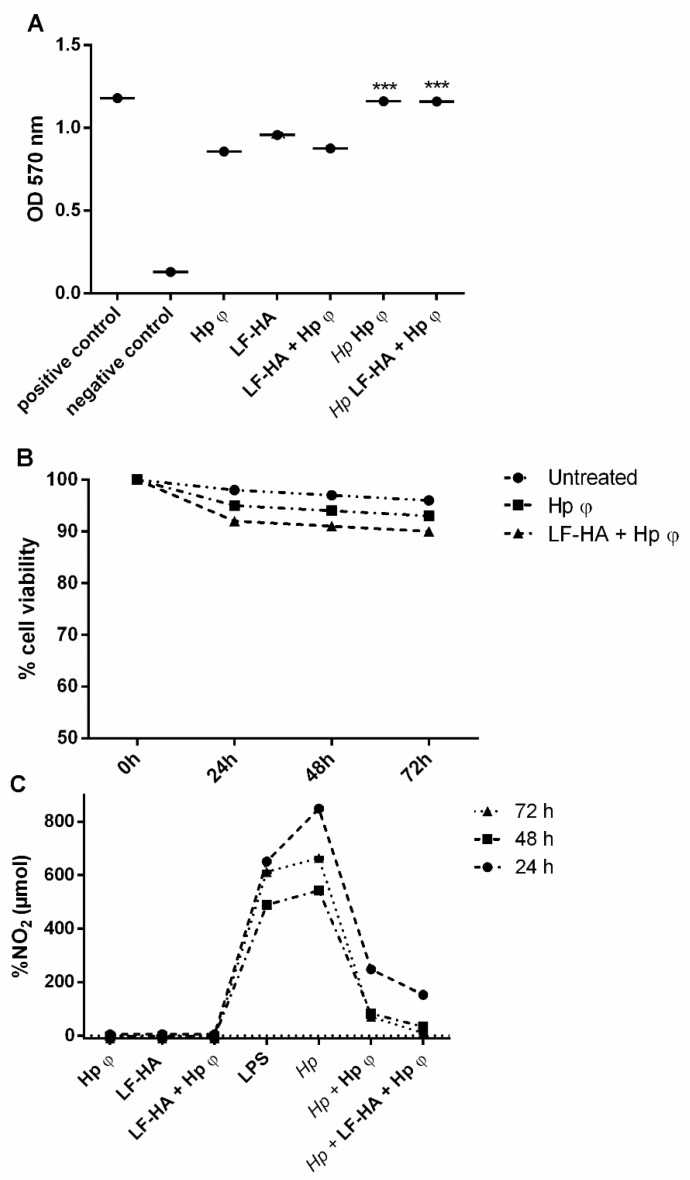
Cytotoxic assays. (**A**) MTT assay for cell viability. The experiment was performed in untreated AGS cells; cells treated with 50% ethanol; phage alone; *Hp* and phage; *Hp* and the complex Hp φ + LF-HA. (**B**) Trypan blue test was performed at 72 h of treatment with 24 h time point. (**C**) % of NO^2^ levels in the culture medium. The measurements, obtained by Griess assay, were carried out in: Untreated AGS cells; cells treated with LPS (10 µg/mL); cells treated with *Hp*; cells treated with phage alone or combined with LF-HA; cells treated with *Hp* and phage alone or combined with LF-HA. *** *p* < 0.001. Statistical analysis was performed with Student’s *t*-test. Values are the mean ± SD from three independent experiments with three replicates for each data point.

The isolated Hp φ tested on human gastric cancer cell lines AGS alone or combined with LF-HA displayed non-cytotoxic activity. AGS cells remained vital for up to 72 h upon the treatments (Figure 3A). These results were confirmed by the Trypan Blue test, showing the treatment with the phage alone or combined with LF-HA does not induce the necrosis of the cells (Figure 3B). Compared to the cells treated with *Hp*, the phage alone or combined with LF-HA reduced NO_2_ production (Figure 3C). In particular, the complex showed the highest reduction of NO_2_ production.

### 3.4. In Vitro Infection of AGS Cells with Helicobacter pylori and Treatment with Hp φ +LF-HA

**Figure 4:**
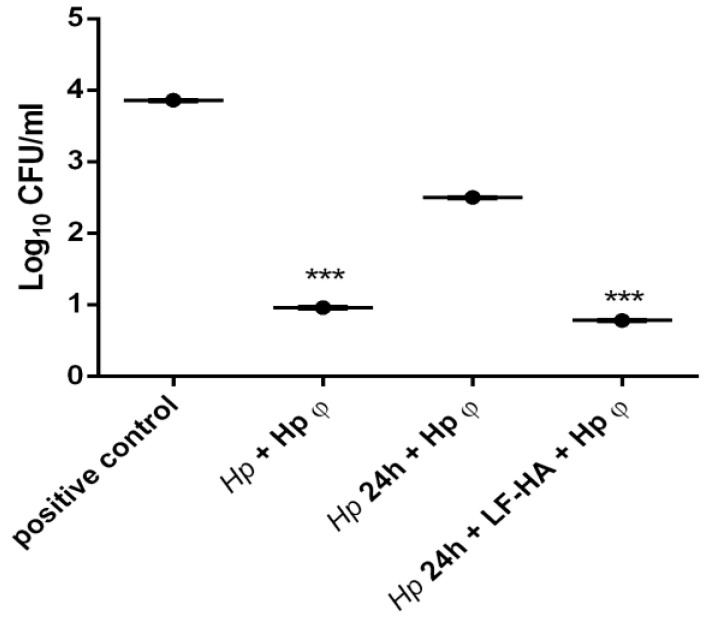
Modulation of antimicrobial effects against *Helicobacter pylori* on AGS cells. Antimicrobial activity of different treatments against *Hp* (10^6^ CFU/mL). Results are presented as mean value ± SD and are representative of three independent experiments, each performed in triplicate. *** *p* value < 0.001.

AGS human gastric cells were infected with *Hp* and incubated simultaneously with the phage; or treated for 24 h with the bacteria and then incubated for 3 h with Hp φ alone or combined with LF-HA. The experiment was carried out in order to understand not only the efficacy of the phage, but the potential application as therapy; for this reason, the lytic activity was analyzed simultaneously or after the infection with *Hp*. The results displayed that the phage exerts antimicrobial activity also when administered when the infection is ongoing (Figure 4). Moreover, we explored the efficacy of the phage combined with LF adsorbed on synthetic hydroxyapatite nanoparticles, administered at 24 h post-infection. The complex displayed a four-fold enhanced antimicrobial activity compared to the phage alone (Figure 4).

**Figure 5:**
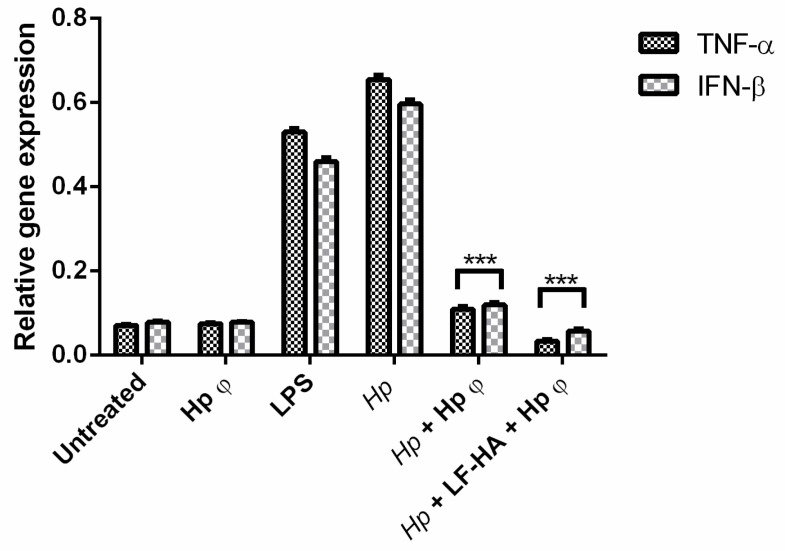
Expression profiling of AGS cell cytokine genes by quantitative real time PCR (qPCR). Cells were collected after 24 h of *Hp* infection and the different treatments. Statistical analysis was performed with Student’s *t*-tests. *** *p* < 0.001.

In particular, Hp φ used for extracellular bacteria reduced the colony counts of *Hp* not only when added simultaneously with the bacterium, but also when it was added 24 h after infection. When in complex with LF-HA, the phage activity can be correlated to the phage co-treatment. This result suggested that when the phage was in complex its activity was stabilized and increased over time. Hp φ +LF-HA performed better also in terms of anti-inflammatory activity, inducing lower levels of *TNF-α* and *IFN-β* genes (Figure 5)

### 3.5. ROS Detection Assay

**Figure 6:**
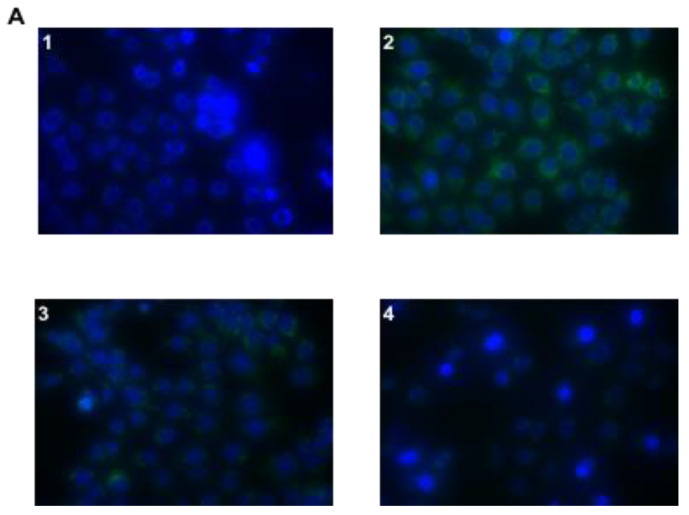

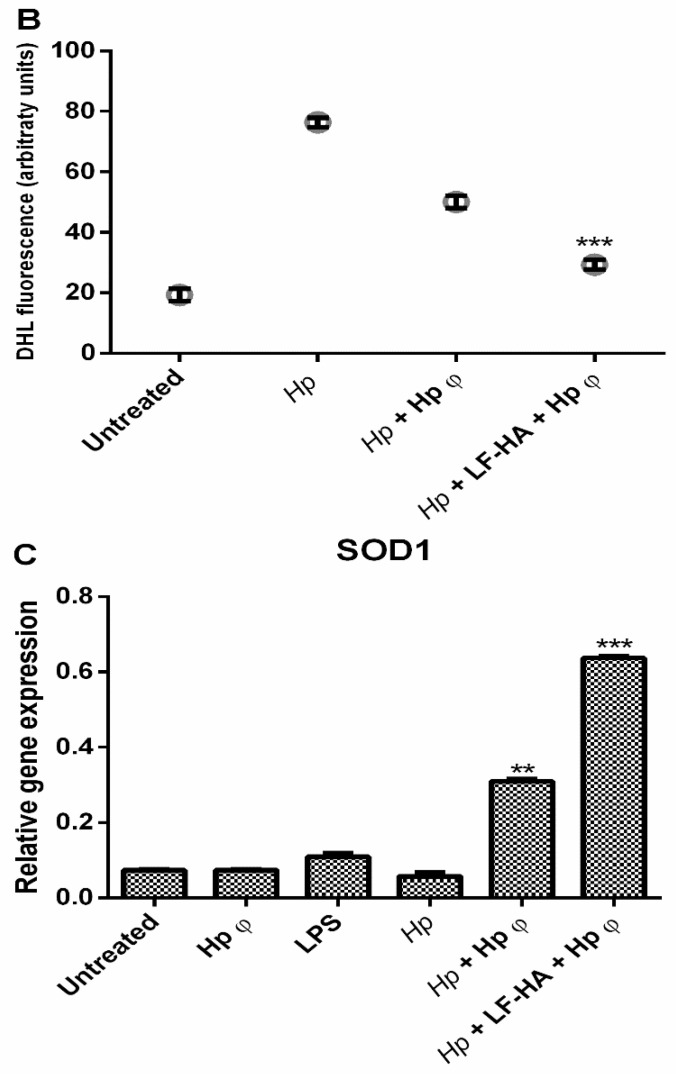
Effect of Hp φ + LF-HA on ROS production. (**A**) DHR-loaded cells differently treated (1 untreated cell; 2 *Hp*; 3 *Hp* + Hp φ; 4 *Hp* +LF-HA + Hp φ), observed with a Leica DMI6000 fluorescence microscope equipped with Metamorph Imaging Software (Leica MetaMorph^©^ AF, Wetzlar, Germany.). (**B**) Quantification of the mean fluorescence of individual cells. Results are expressed as arbitrary units and represent the means ± SD calculated from three to five separate experiments, each performed in duplicate. (**C**) Relative gene expression of *SOD1*. Statistical analysis was performed with Student’s *t*-tests. ** *p* < 0.01; *** *p* < 0.001.

Fluorescence microscope examination identified ROS overproduction in AGS cells infected with *Hp*, compared to control cells and a reduced production of ROS in cells treated with *Hp* phage, compared to *Hp* infected cells (Figure 6). A stronger reduction of ROS production was detected in cells treated with the complex Hp φ + LF-HA compared to cells treated with Hp φ (Figure 6A). In Figure 6B, results are reported as quantification of fluorescence of individual cells. To validate the ROS analysis the quantification of SOD1 production was carried out (Figure 6C). The relative gene expression confirms the decrease of ROS production when the AGS cells were treated with Hp φ + LF-HA more that the cell treated with Hp φ alone.

## 4. Discussion

The long-term *Hp* infection, and the associated chronic inflammation are the main risk factors for gastric cancer and extra-gastric diseases, which have an important social and economic impact worldwide, as they affect more than 50% of the population. Consequently, the treatment of *Hp* infection is essential for the management of more severe secondary pathologies. The recent and ever increasing problem of antibiotic resistance has determined the failure of the conventional treatment (proton pump inhibitors combined with clarithromycin and amoxicillin or metronidazole) against *Hp* infection [36,37]. Encouraging data are reported for the single three in one formulation, of metronidazole and tetracycline combined with bismute subcitrate potassium, which is able to eradicate *H. pylori*–clarithromycin resistant strains [38]. However, the urgent need of treatments able to substitute for common antibiotics makes the identification of innovative therapies necessary.

In previous studies, we showed the effective antimicrobial role of LF adsorbed on hydroxyapatite nanoparticles in *Hp* infection [23]. LF is an iron-binding protein able to chelate iron, inhibiting bacteria growth, and thus displaying bacteriostatic properties. In addition, lactoferrin binds to the outer membrane of Gram-negative bacteria, promoting the release of lipopolysaccharide (LPS) and cell lysis, thus also acting as a bactericidal [39,40].

In the present study, for the first time, we explored the role of *Hp* specific lytic phage (Hp φ) alone or combined with lactoferrin adsorbed on hydroxyapatite nanoparticles (Hp φ +LF-HA) in counteracting *Hp* infection. We prove that LF-HA significantly increases Hp φ activity. Our study suggests that phages complexed with lactoferrin are a powerful biological tool, able to specifically kill *Hp* without toxic effect on the host cells [41], thus representing a good therapeutic strategy in *Hp* infection. Moreover, the use of hydroxyapatite as carrier, able to improve the biological properties of both Hp φ and LF [21,33], further supports our data. The efficacy of the above-described treatments was tested by using an in vitro model of *Hp* infection. In AGS cells infected with *Hp* for 24 h we found five-fold enhanced antimicrobial activity of the complex Hp φ +LF-HA compared to Hp φ (Figure 4). In fact, the complex Hp φ +LF-HA administered after the infection was observed to maintain the bacterial load at the same level measured in cells infected with *Hp* and simultaneously treated with Hp φ (Figure 4). These data indicate that LF-HA potentiates the antimicrobial activity of Hp φ.

We next investigated the anti-inflammatory activity of Hp φ alone or combined with LF-HA. Inflammation plays a key role in *Hp* infection by establishing serious tissue damages if not contained [42]. Both phage and lactoferrin have been recognized as modulators of inflammation, by attenuating the activation of the transcription factor NF-kB and in turn the release of pro-inflammatory cytokines [26,43]. The reduction of pro-inflammatory cytokines can be related to the LPS-binding properties of LF and phage, preventing LPS interaction with Toll-like receptor 4 (TLR4).

Based on this finding, we evaluated the expression level of gene encoding for TNF-alpha and IFN-beta, the main cytokines produced by LPS-stimulated TLR4 activation [44,45]. Cells treated with Hp φ +LF-HA showed reduced TNF-alpha and IFN-beta gene expression, compared to cells treated with Hp φ alone (Figure 5). Interestingly, we observed that the expression level of TNF-alpha and IFN-beta genes was lower in Hp φ +LF-HA treated cells than in untreated ones (Figure 5).

A further confirmation of the role of phage and lactoferrin in modulating the inflammatory response was obtained by evaluating their capability of a negative regulation of *H. pylori*-induced reactive oxygen and nitrogen species (ROS and RNS) formation. In the presence of *H. pylori*, gastric epithelial cells produce ROS and RNS, an important hallmark of inflammation, which can lead to macromolecule damage, thus resulting in detrimental effects for the host cells rather than the microbial ones [46,47]. In the presence of Hp φ, we detected a mitigated accumulation of ROS in infected AGS cells (Figure 6A,B). On the other hand, Hp φ + LF-HA treatment considerably reduced ROS accumulation, compared to both Hp φ treated and untreated cells (Figure 6A,B). Similar data were also obtained for the reactive nitrogen specie NO_2_ (Figure 3C). In addition, we examined the expression levels of the *SOD1* gene, encoding for the antioxidant SOD1 enzyme, involved in neutralizing ROS and RNS [48]. An increased level of *SOD1* gene was found in Hp φ +LF-HA treated cells (Figure 6C), suggesting that the complex Hp φ +LF-HA protects epithelial cells against the *H. pylori*-induced oxidative stress, by up-regulating the expression of antioxidant species [49].

## 5. Conclusions

In conclusion, this study describes the synergistic action of Hp φ and LF-HA against *H. pylori*, and their capability to act as direct or indirect antimicrobial agents by reducing the bacterial colonization and the associated inflammation. However, some limitations should be noted. One of the major advantages of a combined therapy, both safety-wise and cost-wise, consists in reducing the doses of the single treatments. We addressed the potential therapeutic applicability of the complex Hp φ +LF-HA, proving its effectiveness when administered upon the onset of the infection, but we did not determine the minimal effective combined doses of Hp φ and LF-HA. We also still need to determine the genetic barrier to bacterial resistance against the complex Hp φ +LF-HA. Lastly, additional in vivo studies should be performed in order to further investigate the efficacy and the clinical potential of the combined therapy Nonetheless our findings suggest the complex Hp φ +LF-HA as an innovative therapeutic approach for *Hp* infection. Importantly, this work may pave the way for a novel class of combined antibacterial therapies designed to fight a wide range of gastric infections.

## Figures and Tables

**Table 1:** Phage host range determination on different samples from the gastroenterology department of “Ospedale Evangelico Villa Betania” Naples.

*Hp Samples*	Hp φ
Patient 1	-
Patient 2	+
Patient 3	+
Patient 4	+
Patient 5	+
Patient 6	+
Patient 7	+
Patient 8	+
Patient 9	+
Patient 10	+
Patient 11	+
Patient 12	+
Patient 13	+
Patient 14	+
Patient 15	-
Patient 16	-
Patient 17	+
Patient 18	+
Patient 19	+
Patient 20	-
Patient 21	-
Patient 22	+
Patient 23	+
Patient 24	+
Patient 25	+
Patient 26	+
Patient 27	+
Patient 28	+

-, negative lysis result; +, positive lysis result.
